# Missing topics for a newly established general practice curriculum for medical students in Hesse – a qualitative study

**DOI:** 10.1186/s12875-024-02533-y

**Published:** 2024-08-15

**Authors:** Bibiane Kronemann, Elisabeth Joson-Teichert, Matthias Michiels-Corsten, Stefan Bösner, Jana Groth

**Affiliations:** Department of General Practice and Family Medicine, Faculty of Medicine, Philipps-University of Marburg, Marburg, Germany

**Keywords:** General practice, Primary care, Curriculum, Extracurricular teaching, Teaching methods, Qualitative research

## Abstract

**Background:**

To address the declining numbers of general practitioners (GPs) in rural areas and a lack of medical students pursuing a career in primary care, a general practice-based curriculum coupled with additional university admissions for students has been established at three universities in Hesse, Germany. This study aims to analyze potential topics which students striving to become a GP will benefit from. Teaching such topics will prepare them for their chosen career and working in rural areas. We aimed to explore the views of both specialists and GPs on chief topics and necessary skills in primary care.

**Methods:**

In our study we used semi-structured interviews with outpatient specialists and specialists in clinical practice and semi-structured group interviews with GPs in training. The topic guide addressed contents of the curriculum for medical students with an extracurricular focus (addressing additional topics) on primary care. Data analysis was carried out using qualitative content analysis according to Mayring.

**Results:**

GPs in training and specialists agreed on the importance of knowledge in the fields of medical history, physical examination, communication as well as common diseases in primary care. Essential competences mentioned were: inducing medical treatment, decision-making and triage, conducting structured conversations, having patient knowledge (hard skills) as well as an interest in continuous learning, empathy, personal commitment, listening and down-to-earthness (soft skills). Case reports, symptom-based learning, practical training, lessons with simulated patients and the integration of role models were regarded as useful teaching methods.

**Conclusions:**

General practice-based curriculums should not only focus on the transfer of knowledge. Equally important is the training of soft and hard skills to prepare future GPs for their work in primary care. Special teaching methods as well as practical training should be the heart of a newly established curriculum.

**Supplementary Information:**

The online version contains supplementary material available at 10.1186/s12875-024-02533-y.

## Background

Recruitment of general practitioners (GPs) in Germany has become a great political concern as rural areas are expected to be severely understaffed by 2030 [1, 2]. To address the lack of GPs in Germany, a separate admission system for students starting medical school has been established in three universities in Hesse (Universities of Marburg, Frankfurt and Giessen). Since October 2022 6,5% of the newly assigned medical students at these universities have received admission into medical school with the condition to pursue their future careers as GPs or pediatricians (“Landarztquote”). Students are bound by contract to work in general practice or pediatrics for at least 10 years and will receive special extracurricular training during their studies at Hessian Universities – this comprises a seminar and mentoring program including practical training in rural areas [3]. Regular students who obtain a university placement without this quota are also free to partake in the extracurricular teaching program without a contract to pursue work in primary care. Therefore, the teaching program consists of two aims: (1) to best prepare the students of the quota for their chosen career and working in rural areas; and (2) to interest other students in primary care.

In Germany, the GP obtains the primary role in sorting a patient’s way through specialist consultations and clinic visitations. This so-called “Lotsenfunktion” role is designed to improve the patient-physician relationship. Moreover, it helps in guiding patients in the health care system and reducing the amount of unnecessary specialist consultations through a first-line check by the GP [4].

Various studies have discussed why only a few students are interested in primary care and how to improve interest and recruiting students for primary care. Key findings include improving access to internships and learning practical skills in undergraduate studies [5], reducing the lack of visibility and physicality of what GPs really do and tackling stereotypes such as gender stereotypes or the stereotype of income inequality between other specialties and general practice [6]. Additionally, there is a strong call for teaching on economic aspects of primary care [7]. Students furthermore described the lack of role models in general practice [8].

There are many studies on what topics and competences should be included in a general practice-based teaching curriculum [9–13], such as a competence-based approach [11], including an app in teaching [12] or interprofessional education to improve teamwork abilities [13]. Other studies found support for the effectiveness of longitudinal, multifaceted teaching programs in primary care [14], suggested a special educational program with an orientation towards rural primary care [15] or stated the importance of a long-term one-on-one mentoring program [16].

Briefly, the existing research on teaching programs in primary care calls for longitudinal, mentor-based teaching courses along with practical training and positive role models in primary care [7, 11, 12, 14, 17].

To develop a longitudinal general practice curriculum at the University of Marburg we interviewed specialists and GPs in training on topics and skills which need to be taught to prospective GPs. To put it into context, this study is part of a larger study, where we interviewed experienced GPs and students as well. All four named groups will be of importance for the design of the curriculum, however, we decided to focus on the two named groups here as they have not been interviewed in other research projects. Some comparable projects of longitudinal teaching have been discussed and set in place throughout Germany during the last years [7, 14, 16–21]. However, for the development of these programs – if at all – only students and GPs have been consulted [9–13]. Through integrating the views of specialists and GPs in training we hoped to discover new findings to broaden our newly established general practice curriculum for medical students in Hesse.

## Methods

### Design of the study

We chose a qualitative approach consisting of ten semi-structured interviews with outpatient specialists and specialists in clinical practice of fields working closely with GPs, namely urology, orthopedics, ophthalmology, otorhinolaryngology and surgery. Furthermore, we performed three semi-structured group interviews with nine young doctors in training for general practice. The research’s theoretical framework and setting allowed us to explore the dynamics of personal experiences in primary care as well as suitable topics and course structures for the newly established curriculum. The group interviews were chosen as an approach for the GPs in training to include group reactions in addition to interpersonal experiences. The qualitative approach aims to illustrate personal views of a small number of specialists and GPs in training to explore the field in greater depth and collect data for a successive quantitative study.

The questionnaire for the interviews was developed specifically for this study and can be found as a supplementary file attached to the study. The piloted questionnaire was developed by BK, ET and MMC. BK interviewed the participants.

Our researchers have no personal relationship with the participants. However, we all work in primary care and have experience with the extracurricular teaching program in Marburg either as students, lecturers, or coordinators.

### Sample

Between April 2022 and February 2023, we recruited specialists and GPs in training interested in participating in our study. Recruiting was based on personal interactions in hospitals and doctors’ offices in Hesse. Furthermore, we published advertisements for the study in a network of GPs in training in Germany and Hesse and on social media (namely X, formerly Twitter).

Ten specialists and nine GPs in training expressed interest in taking part in the study. All of them were included in the final interviews. After three sets of group interviews (GPs in training) and ten individual interviews (two per specialty) thematic saturation was achieved.

**Table 1:** Sample of interview participants (*n* = 19)

**Gender**	
Male Female	*n* = 14 *n* = 5
**Age**	mean = 43,5 years min. 30 years max. 69 years
**Number of years in practice**	
< 10 years > 10 years	*n* = 6 *n* = 13
**Practice type**	
Group practice Solo practice	*n* = 14 *n* = 5
**Practice location**	
Rural area (< 5.000 habitants) Small town (5.000–20.000 habitants) Medium-sized town (20.000-100.000 habitants) Large town (> 100.000 habitants)	*n* = 2 *n* = 6 *n* = 9 *n* = 2

### Data collection

The interviews were undertaken in the practice of the specialists or online via a secure web tool, whereas all group interviews were undertaken online. The interviews were semi-structured and lasted between 30 and 90 min. All interviews were recorded digitally and transcribed verbatim.

The interview guide focused on views and experiences related to central topics and necessary skills taught to promote optional preparation for work as a GP in rural areas.

The aims of the study were explained to each interviewee and it was ensured that all questions were sufficiently explained to avoid misunderstandings.

The instruments for data collection did not change during the conduction of the interviews. The interviews took place between June 2022 and February 2023.

### Ethics approval

The ethics committee of the University of Marburg approved our study design. The ethics approval was assigned on the 28th of April 2022 (ethical approval number 52/22).

### Data analysis

**Fig. 1:**
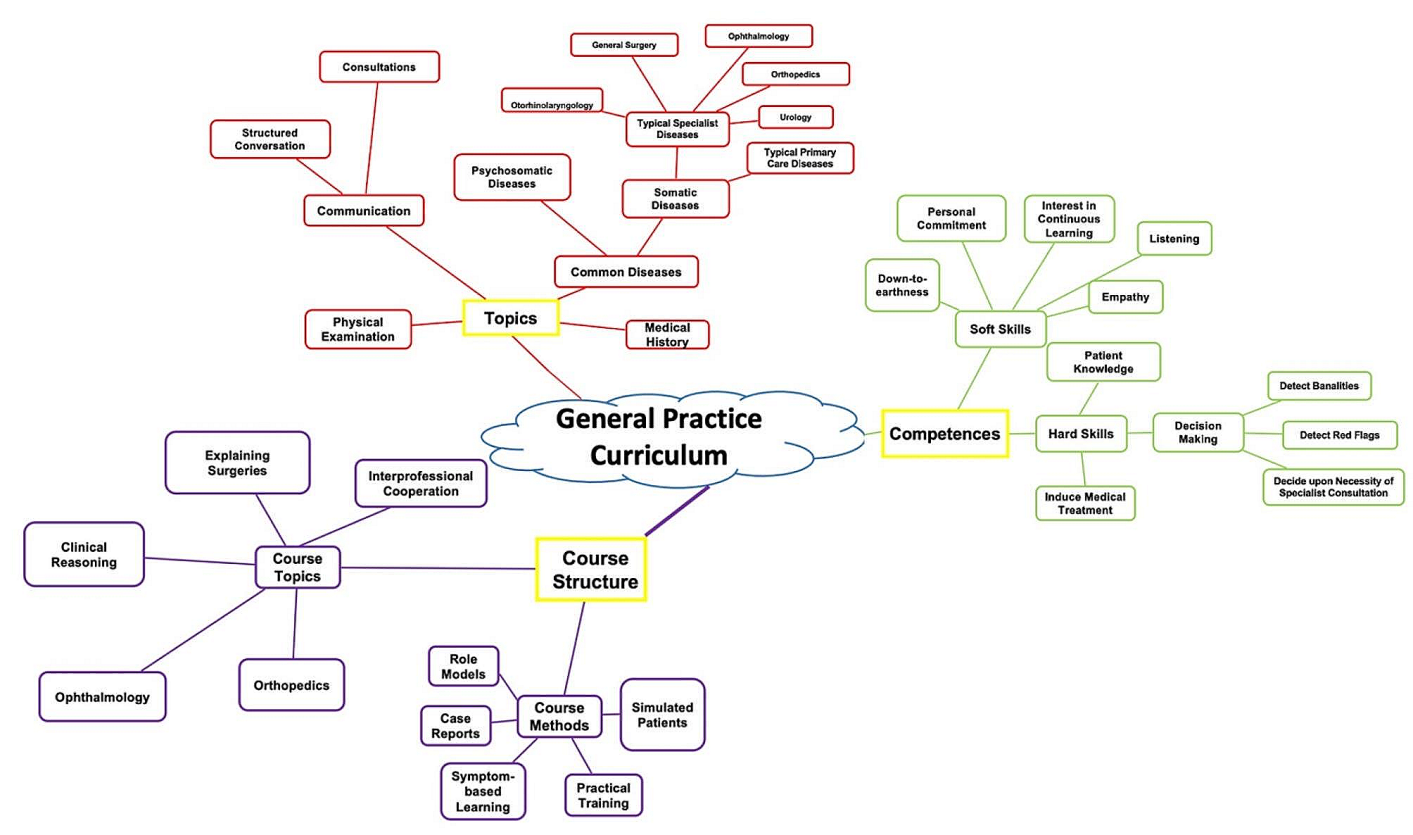
Code structure with main and sub categories

The interviews were carried out between June 2022 and February 2023. The analysis was performed using the software MAXQDA2022. We identified key issues and named codes which were sorted into main categories and sub categories (see Fig. 1) on the basis of P. Mayring’s qualitative content analysis [22]. The three main categories (topics, competences, course structure) were developed deductively based on the interview guide. All sub categories were developed inductively. To test interrater reliability four content-rich interviews (two group interviews and two single-person interviews) were exchanged between the authors. Afterwards, the code system was slightly adapted. Interviews and analyses were conducted simultaneously to optimize substantial saturation by the researchers. BK and EJT independently reviewed transcripts in confirmation of comprehension and reproductivity of codes applied. Any disagreements were discussed, and a consensus found. Cited quotations were translated by BK from German into English and cross-checked by JG.

We identified three main categories in the transcripts: ‘topics’, `Competences´ and `Course structure´. We defined **topics** as contents or subjects that should be included in the curriculum. A typical quote from the interview would include “I would like a seminar on …”. We as researchers asked ourselves “Can we know this?” to determine answers coded into this category. The second main category, **competences**, was defined as skills or abilities the students and our curriculum require. In an interview the competences would be described as “The doctor has to be able to …”. Accordingly, we asked ourselves “Can we do this?” to verify our coding. The third main category, **course structure**, included specific proposals and recommendations for teaching methods and course formats.

## Results

### Topics

GPs in training and specialists noted communication being a central topic for students, especially structured conversation, and consultation in primary care.

*“And it’s not just about how do you do a good medical history*,* but also how do you handle someone who cannot get to the point? How do you treat people blowing up your office hours with their consultations? Not just this part of medical history*,* where you ask when it started and where the pain is located. But also what do I do with someone who does not stop talking?” (F-4-CO)*.

Furthermore, a recurring topic requested by interviewees was common or widespread diseases in primary care. Psychosomatic diseases were mentioned here more often.

*“When I started and my boss told me two-thirds of consultations are of a psychic or psychosomatic background*,* I could not believe it really. But it is a lot*,* and you have to deal with it.” (F-7-UK)*.

For somatic diseases, common diseases in primary care – such as back pain, high blood pressure or diabetes – including typical specialists’ diseases were mentioned. In the case of ophthalmology, this was a cluster of diagnoses referring to the “red eye”, chronic or acute loss of sight as well as high sensibility towards light. Furthermore, the surgeons named hemorrhoids, acute abdomen and wound healing deficiency. Orthopedists described back pain to be common, as well as joint infections and traumatic injuries. Urologists mentioned bloody urine, urinary retention, urinary stones, and prostatic diseases to be a recurring topic in primary care. Conversely, otorhinolaryngologists named ear pain, upper respiratory infections, sudden loss of hearing and other emergency treatments.

*“And concerning treatment or primary care of various clinical pictures*,* I believe the emergency treatment of this specialty needs to be a part of university teaching because it also defines the intersection with other specialties. Even if you won’t be an otorhinolaryngologist you should definitely know how to treat a nose bleed*,* which are the key symptoms of an otitis media or a peritonsillar abscess or a beginning mastoiditis.“ (P-6-MS)*.

Most interviewees described medical history and physical examination to be important aspects of existing curricular teaching. Yet, they appealed for further highlighting and inclusion in extracurricular teachings.

*“And medical history before the physical examination. (…) How can you reach the core of the problem with focused questions in a short timeframe? Here you can get lost easily and imagine the worst. But maybe you can break down the contact to mostly 10 or 15 minutes.” (F-6-JM)*.

### Competences

Concerning competences, hard skills as well as soft skills were mentioned in the interviews. Soft skills is a term we use for general personal and social competences, whereas hard skills refer to specific professional competences of a GP.

Regarding soft skills, the interviewees considered empathy, personal commitment, listening, down-to-earthness and interest in continuous learning as important factors in primary care.

*„A GP must be humane. He has to care. In the end the patient must have the feeling that he is in good hands.” (P-7-SGa)*.

All interviewees also emphasized the importance of hard skills. Recurring topics were decision making and triage in primary care under the condition of a limited timeframe for consultation, which results in high need for attentiveness in patient care. The main hard skills mentioned were to decide upon the necessity of specialist involvement, to detect banalities easily treated in practice instead of sending them to a special clinic with long wait, and to detect red flags and dangerous aversive outcomes.

*“But this is a singular outstanding attribute of primary care. We are gatekeepers. Most patients we see do not have the most severe progression or any alarming red flags. But to sieve those that do*,* that is our duty. (…) The majority has a cough or cold and want a sick leave from work. And those patients don’t believe their sickness to be threatening and neither do I*,* yet it is self-evident to check for any red flags nonetheless. And then the patient is content and happy to go home. But to always stay alert.” (F-3-FR)*.

Equally important hard skills in primary care were considered to be being able to induce medical treatment and having good patient knowledge (including an understanding of their living situation and their medical knowledge).

*“Well*,* I would say he needs to know his people. That is something I know and value about good general practitioners in my environment. They can assess common settings and I think that is central*,* or important*,* to know where your patients are coming from. In cities that is a bit harder nowadays*,* compared to rural areas where you know the family and understand the context. But for me it is a very important aspect. To be able to assess a situation. Where does it come from? What is the context? This is a quality I would appreciate in a general practitioner.” (P-8-JBe)*.

### Course structure

The interviews showed differences in the course topics described, as specialists naturally described mostly topics of their subject to be of importance for GPs. This entailed an orthopedics course or a seminar on the red eye. One of the interviewed surgeons also suggested lessons on explaining important surgeries to patients. This course would, inter alia, help a GP explain not only the methods with which a specialist will treat an illness, but also help the GPs explain the consequences of a surgical treatment.

An interest in interprofessional cooperation as a course topic was expressed by one of the specialists.

One participant mentioned an existing course at the University of Marburg in which case reports or common diseases are worked out in groups to describe evidence-based treatment options and differential diagnostic procedures.

*“Clinical Reasoning (…) A case report is presented together with differential diagnoses and therapeutic options. The case is not necessarily clarified in the end. You just follow. Well*,* the teacher has to vary according to what the students answer and it is (…) You start with chest pain and in the end*,* you get the idea of gastritis as a diagnosis and then you discuss the treatment options. It’s an interactive course and I loved it. You think about the case as one would in practice. And that’s the closest we can get to a first line patient consultation in primary care.” (F-1-HU)*.

Besides course topics, specific teaching methods were named. This included popular methods already existing in teaching modules in university, such as case reports, symptom-based learning and practical training. This includes internships as well as learning practical skills at university.

On top of that, several GPs in training agreed that especially lessons with simulated patients had been a helpful method in their studies.

*“You must stay flexible in your thinking (….) A lesson with simulated patients and then there can be anything happening. To have patients that are not selected by subject beforehand. One has an itch without a cause. Another patient is a one-year-old infant with a fever. Next*,* there’s the 90-year-old patient who hasn’t had a doctor’s visit in ten years and is to be treated palliatively. To portray this in university. A course with simulated patients that is as messy as family practice.” (F-1-HU)*.

In two interviews, interviewees described how role models in primary care influenced their career paths and expressed interest in implementing this into the curriculum.

*“And what I’ve always found especially exciting were the couch talks*,* where a random doctor came to talk about everyday life in practice. To me these personal topics were extremely important to have a role model. Because with many specialties I thought: ‘Oh god*,* if you end up like this it’s over!’ And in general practice*,* I met people where I thought: ‘Hey*,* this is pretty cool.’ And they invited me out for lunch during the internships and I thought that was… Well*,* the personal aspect is so important to hang on. To have these role models and to get an insight beyond professional aspects to stay motivated*,* to say ‘I want to do this too.’” (F-3-FR)*.

## Discussion

### Key findings

There was fair distinction in topics named or highlighted by specialists and GPs in training. This ties in with the interview guide where specialists were mostly asked what part of their subject need to be represented and taught in the curriculum, while GPs in training were asked about their experiences in practice.

Referring to course structures, particularly the GPs in training named already existing courses they remembered to be of essence to their personal career and education. Specialists described ways to communicate the essence of their subjects in a limited format taking into consideration the limitations of extracurricular teaching modules and the main interest of the curriculum being primary care.

Throughout all interviewees, there was great consensus on a high demand for the discussed competences, with special highlight for decision-making in primary care. This was explained by the changing role and demand of GPs as an understaffed specialty and high stress from the concurring factors of limited time for consultations as well as a high number of patients and workload per day.

Numerous aforementioned issues are already part of the existing curriculum (e.g. courses on common diseases, communication, decision making, empathy, clinical reasoning, interprofessional cooperation as well as the use of role models, case reports and symptom-based learning). Other aspects (e.g. courses on explaining surgeries, ophthalmology, orthopedics) are not included in the curriculum. Also, the use of simulated patients and the amount of practical training could be increased. This will all be the subject of future discussions.

### Strengths and limitations

The study includes a mixed sample balancing demographic characteristics such as gender and age as well as working conditions such as solo or group practice and working in a rural area, small or medium-sized town. Nonetheless, we undertook the study in only one region of Germany with a limited amount of interviewees. Findings may not be transferable to other regions of Germany or fit the impressions of the majority of GPs in training or specialists.

A strength of the study can be described by a trustful setting for the conducting of interviews thereby reducing the impact of social desirability and presumably producing authentic reports on opinions and experiences of the interviewees.

Another strength of the study is that we did not only include the perception of GPs in training but also that of specialists working closely with GPs. Thus, we include not only the individual perceptions of their own profession but also external input and feedback on necessary knowledge, competences and teaching courses.

For our main research question “Which topics are missing from the general practice-based curriculum?” both GPs’ in training as well as specialists’ viewpoints have proven highly relevant.

### Comparison with existing literature

Similar to previous studies [5, 6, 9] we found that internships, practical training, interprofessional education, and the integration of positive role models were considered as highly important for the development of a general practice-based curriculum. GPs in training and specialists agreed on the importance of knowledge in the fields of medical history, physical examination, communication as well as common diseases in primary care. In addition, the interviewees emphasized the relevance of competences (soft as well as hard skills) to supplement theoretical knowledge, confirming the idea of a competence-based curriculum mentioned in the literature [11]. Moreover, similar to the suggestion in literature [8], it was emphasized that the implementation of role models and personal insights helps to increase the appeal of general practice to students. Other recommendations – like the importance of a long-term one-on-one mentoring program [16] or the integration of a teaching app [12] – were not mentioned in our interviews. A new insight is the demand for special teaching methods such as symptom-based learning, lessons with simulated patients and the integration of case reports.

Diverse studies have proven problem-based-learning (PBL) and case-based-learning (CBL) to have positive effects on theoretical knowledge and patient care [23], longer-term knowledge retention and application of knowledge [24], patient assessment [25] as well as communication skills, problem-solving ability, and learning motivation [26, 27]. A combination of PBL and CBL increases scores in several fields such as learning motivation, understanding, student-teacher interaction, the final examination, communication skills, clinical thinking skills, self-learning skills, team working skills, or knowledge absorption [28]. However, a systematic review on the use of simulated patients in medical education has suggested that there is no superiority of this teaching method in comparison with traditional teaching methods [29]. Therefore, we suggest to focus on the integration of PBL and CBL such as symptom-based learning and case reports when implementing special teaching methods into a primary care teaching program.

## Conclusions

Our study has proven to show valuable input in the research on longitudinal teaching courses nationally and may be able to add valuable insights to teaching programs internationally. According to GPs in training and specialists, soft and hard skill training will be equally important to prepare future GPs for their work in primary care as classic teaching of theoretical knowledge. GPs in training and specialists also agreed that special teaching methods as well as practical training should be the heart of a newly established curriculum.

Further studies should integrate the views of medical students as well as experienced GPs in order to compare the perspectives of medical students, GPs in training, experienced GPs and specialists. Furthermore, a quantitative study may help evaluation and prioritization of the mentioned topics, skills and teaching methods by a larger number of medical students, GPs in training, experienced GPs and specialists.

In a successive quantitative study we plan to examine whether these findings are supported by a larger number of GPs in training. We are currently also conducting a complementary qualitative study on the views of medical students and experienced GPs. Depending on those outcomes of the two studies, we will decide how to revise the existing curriculum.

### Electronic supplementary material

Below is the link to the electronic supplementary material.

Supplementary Material 1

## Data Availability

The datasets analyzed during the current study are not publicly available due to safeguarding of personal information and privacy of participants but are available from the corresponding author on reasonable request.

## Abbreviations

GP: General Practitioner
