# Supplementary material for: Missing topics for a newly established general practice curriculum for medical students in Hesse – a qualitative study
Source: BMC Prim Care. 2024 Aug 15;25:306. doi: 10.1186/s12875-024-02533-y (PMC11325750; doi:10.1186/s12875-024-02533-y)
Supplement: Supplementary file 1 — Supplementary Material 1 [file 12875_2024_2533_MOESM1_ESM.docx]

Questionnaire – Guideline Single Person Interviews with **Outpatient Specialists**

**Personal Background**

- What led you to work in the position/specialty you are in today?

**Intersections/Cooperation**

- How do you view cooperation of your practice/clinic with colleagues in primary care?
- On which occasions did you communicate with general practitioners (in)directly within the last weeks/months?
- In what way are you in contact with rural general practitioners in your environment?

**Communication**

- How would you describe the need for general practitioners in the rural area where you work?
- Are referrals indicated and right? Does the preselection work well, meaning that you don’t see patients who wouldn`t have needed a specialist consultation?
- How can we reduce this in the future? Do you communicate this problem with the transferring general practitioner?

**Qualities/Competences**

- What defines a good general practitioner in rural areas?
- Which personal competences does a doctor need to communicate and interact with patients?
- Which medical competences does a doctor need in primary care? Which of these can be taught in university already?
- Which competences are more important to you? Why?

**Exploration**

- Do you have an exact idea in mind, what we should definitely include in the curriculum for future general practitioners?
- Can you imagine partaking in the teaching of students of the longitudinal teaching program? If yes, how?
- What would you wish for if you were to study again today?

Questionnaire – Guideline Group Interviews with **General Practitioners in Training**

**Introduction**

- Warm-up-Questions:
  - Why do you work in primary care?
  - What situation in the last month/week was representative of working in primary care to you? Did you perceive this situation positively or negatively?

**Discussion**

- Open Questions:
  - What`s your experience with…
    - …preparedness for work life through university?
    - …learning about interdisciplinary cooperation in university?
    - …developing competences in university?
    - …mentoring in university?
- Competences:
  - What defines a good general practitioner in rural areas?
  - Which personal competences does a doctor need to communicate and interact with patients?
  - Which medical competences does a doctor need in primary care?
  - Which competences are more important to you? Why?
- Interdisciplinary Cooperation:
  - What is your experience with interdisciplinary cooperation (hospitals, practices, other areas like physiotherapists etc.)?
  - What would improve cooperation? What can you learn about this in university?
  - Do you feel like you were prepared well for this work in university? Why?
  - How do you feel about communication in primary care? How can we improve this? Is this a topic for university teaching?
  - Did you receive criticism or help with this during your education?
- Exploration:
  - Which competences/topics are not being taught enough/at all in university?

**Conclusion**

- Last Questions:
  - What do you wish for the longitudinal teaching program?
  - What would you wish for if you were to study again today?
